# Recovery of Extracellular Lipolytic Enzymes from *Macrophomina phaseolina* by Foam Fractionation with Air

**DOI:** 10.1155/2013/897420

**Published:** 2013-05-13

**Authors:** Claudia Schinke, José Carlos Germani

**Affiliations:** Laboratório de Tecnologia Bioquímica, Faculdade de Farmácia, Universidade Federal do Rio Grande do Sul, Avenidal Ipiranga 2752, Sala 709, 90610-000 Porto Alegre, RS, Brazil

## Abstract

*Macrophomina phaseolina* was cultivated in complex and simple media for the production of extracellular lipolytic enzymes. Culture supernatants were batch foam fractionated for the recovery of these enzymes, and column design and operation included the use of P 2 frit (porosity 40 to 100 **μ**m), air as sparging gas at variable flow rates, and Triton X-100 added at the beginning or gradually in aliquots. Samples taken at intervals showed the progress of the kinetic and the efficiency parameters. Best results were obtained with the simple medium supernatant by combining the stepwise addition of small amounts of the surfactant with the variation of the air flow rates along the separation. Inert proteins were foamed out first, and the subsequent foamate was enriched in the enzymes, showing estimated activity recovery *(R)*, enrichment ratio *(E)*, and purification factor *(P)* of 45%, 34.7, and 2.9, respectively. Lipases were present in the enriched foamate.

## 1. Introduction

*Macrophomina phaseolina *(Tassi) Goid. [1], the only species of its gender, is a phytopathogenic filamentous fungus, belonging to the anamorphic Ascomycota, Botryosphaeriaceae family [2]. It was recently described as possessing “tools to kill” [3] due to its genome providing a diversified arsenal of enzymatic and toxin tools to destroy the host plants, a capacity that is confirmed by its ability to infect over 500 different plant species [4]. Thus, to presume that *Macrophomina phaseolina* is able to produce several enzymes suitable for industrial applications is a reasonable hypothesis.

To this purpose, many studies on its cell wall degrading hydrolases were performed [5–12]. However, *M. phaseolina* produces several other extracellular enzymes [13] of potential industrial use, among them lipolytic enzymes, which are excreted into the culture media in different amounts depending on the strain and incubation conditions. No studies were found attempting to purify these lipolytic enzymes.

Several processes in the food industry, as well as environmental and industrial biotechnological applications, use enzymes as biocatalysts [14] due to their many advantages over chemical catalysts: the ability to function under relatively mild conditions of temperature, pH, and pressure; their specificity and, in some cases, their stereoselectivity. In addition, they do not produce unwanted byproducts [15]. Lipases are of particular interest because of their many applications in oleochemistry, organic synthesis, the detergent industry, and nutrition [16], and there is constant search for new options [17].

Eco-friendly technologies are nowadays one of industry's biggest concerns [18]. Foam fractionation, an adsorptive bubble separation technique, has gained attention in recent years as it is environment compatible, cost effective, and a gentle method for the recovery and enrichment of proteins and enzymes [19–24]. Proteins interact with the gas-water interface by means of electrostatic or hydrophobic forces, which promote their selective adsorption on the bubble film according to surface activity characteristics. Those proteins that reduce surface tension will be preferably adsorbed at the interface and concentrated in the foamate, the resulting foam that leaves the foam fractionation column [25], while inactive species remain in the bulk solution.

Most experiments with protein foam fractionation shown in the literature use nitrogen to generate the foam, as its use is believed to avoid oxidation of sensitive molecules [22, 25]. Nonetheless, it was shown that denaturation at the gas-liquid interface is not primarily caused by oxidation, as proven by comparing the results from fractionation with air to those with nitrogen [26]. Studies using air [27, 28] report that recovery depends on the enzyme, pH of the bulk solution, and the gas composition, in which air fares equal or better than nitrogen [29]. 

In foam fractionation experiments with enzymes, usually P 3 frits (porosity 16 to 40 *μ*m) are employed, which produce very small bubbles, favoring protein recovery. However, it has been demonstrated that larger bubbles increase enrichment [30, 31]. Some works, which start from bulk solutions of known protein/enzyme content, show the influence of gas flow rate on enrichment and recovery during batch foaming [32, 33]. No reports were found showing the influence on the efficiency parameters of varying the gas flow rate during a batch foam separation.

Most studies with complex protein mixtures, like culture supernatants, show results without showing the evolution of the process. Besides, when a surfactant was used to help foam fractionate enzymes, the majority of experiments report the addition of a nonnegligible amount of the substance to the initial solution [21, 33, 34], which could render the purified enzymes suitable for restricted applications [35]. Some reports even show that the use of a surfactant denatured the enzyme [28]. 

*Macrophomina phaseolina* produces extracellular lipolytic enzymes in rich liquid culture medium at pH 6.8, reaching peak specific activity in four days [36, 37]. To optimize the conditions for production, in the present work, the complex medium was used at different pH and incubation temperatures. The fungus was also cultivated in a simple mineral salts medium containing Tween 80. The batch foam fractionation of the extracellular lipolytic enzymes of both culture supernatants was attempted. The influence on the efficiency parameters of using a P 2 frit (porosity 40 to 100 *μ*m) and varying the air flow rate during the fractionation was also investigated. Triton X-100 was used, and the effect of its sequential addition was studied.

## 2. Material and Methods

### 2.1. Equipment and Reagents

Reagents and cultivation media were bought from Himedia (India), Merck (Germany), Vetec, and Nuclear (Brazil). Extravirgin olive oil was of commercial grade (La Violetera, Spain), Triton X-100 and Tween 80 from Merck (Germany), Lipozyme TL 100 from Novozymes Latin America Ltda, and bovine serum albumin (BSA) from INLAB Alamar Tecno-Científica Ltda. (Brazil). A Minisart (Sartorius) filter, porosity 0.2 *μ*m, was used for filter sterilizing. The rotatory shaker with controllable temperature was from Oxylab (Brazil), and the Wallac Envision 2104 Multilabel Reader (Perkin Elmer) spectrophotometer was used with the Wallac Envision Manager 1.12 software for microplate readings. The following equipment was also used: a rotameter of 2 to 20 L h^−1^ from Heinrichs Messgeräte (Germany) and activated carbon and sterilizing air filters FR-1200, FTC-1200, and FTA-1200 from BelAir Pneumática & Hidráulica (Brazil).

### 2.2. Microorganism

*Macrophomina phaseolina* isolate MMBF 04-10 was obtained from the Micoteca Mário Barreto Figueiredo of the Instituto Biológico da Secretaria de Agricultura e Abastecimento do Governo do Estado de São Paulo, Brazil. The microorganism was cultivated on potato-dextrose-agar plates, and mycelium discs of 0.5 cm in diameter were cut out and kept in sterilized water for future inoculations.

### 2.3. Influence of the Medium pH and Composition, the Incubation Temperature and Time on the Extracellular Lipolytic Activity of *M. phaseolina*

The microorganism was cultivated in a complex medium consisting of (per liter) 20 g Bacto Proteose Peptone nr. 3, 0.6 g MgSO_4_, 1.0 g KH_2_PO_4_, and 1.0 g (NH_4_)_2_HPO_4_, and pH was adjusted to 4.5, 5.5, or 6.5 with 1 M HCl prior to sterilization. Filter sterilized olive oil (0.8% v/v) was added, as well as three mycelium discs per 100 mL of medium. Incubation at each pH was done at 25°C, 30°C, and 35°C for 96 h in a rotatory shaker at 160 rpm. The samples, taken at 24 h intervals, and the final broth were filtered under vacuum and stored at −17°C until analysis. 

Production of extracellular lipolytic enzymes was also tested in a simple medium containing no protein, consisting of (per liter) 2.0 g NH_4_NO_3_, 2.0 g K_2_HPO_4_, 1.0 g KH_2_PO_4_, 0.2 g MgSO_4_, 0.14 g CaCl_2_, 0.2 g NaCl_2_ [38], and 0.4% (v/v) Tween 80, pH 5.5. The simple medium was added of trace elements solution (2 mL L^−1^) consisting of (per liter) 0.005 g CuSO_4_, 0.08 g FeCl_3_, 0.09 g ZnSO_4_, and 0.03 g EDTA [39]. Three discs of mycelium were added per 100 mL of medium and incubated at 30°C for 96 h at 160 rpm. The samples, taken every 24 h, and the final broth were treated as described for the complex medium.

### 2.4. Foam Fractionation

*Culture Conditions*. For foam fractionation purposes, the microorganism was cultivated in the complex and simple media at pH 5.5, 30°C at 160 rpm for 72 h, and the broth was filtered as mentioned earlier. The culture supernatant from the complex and simple media showed pH of 6.3 and 4.3, respectively. 

*Adjustment of pH*. Culture supernatant was subjected to isoelectric precipitation tests using buffers at different pH, and precipitates obtained at pH 4.4 and in the range of pH 5.6 to 7.0 showed higher lipolytic activities. The pH of the complex medium was adjusted to 5.8 with HCl 0.1 M before foaming. 

*Equipment and Experimental Procedure*. Foam fractionation glassware was similar to that described elsewhere [35], except for using a P 2 frit (porosity 40 to 100 *μ*m) and a 300 mL drainage bowl (diameter, 8.4 cm). Filter-sterilized air from a compressor was let through the frit and bubbled in the liquid, generating a column of rising foam. The drained foam leaving the top of the column (foamate) was collected in a receiving flask. Triton X-100 was added to culture supernatants (100 mL) before fractionation, and all experiments were done at room temperature (23–25°C). Foamate samples were collected at different intervals and allowed to collapse at 4°C before analysis. Protein content and lipolytic activity were determined in the initial solution and foamate samples.

### 2.5. Lipolytic Activity

 For the qualitative assay, rhodamine B plate method [40] containing 0.8% filter sterilized olive oil was used to verify the hydrolytic activity on normal chain triacylglycerols of 10 *μ*L foamate samples showing the highest purification factor. Lipozyme TL 100 was used as positive control. Plates were incubated at room temperature for 4 days. The development, around the inoculation well, of a yellow-orange halo against a pink background when visualized under 350 nm UV light denotes triacylglycerol hydrolysis. Each sample was tested in duplicate.

For quantitative assay, 4-nitrophenyl palmitate (pNPP) was used as substrate using Tris-HCl buffer, pH 8.5, and 30 min incubation at 45°C. Absorbance was read at 410 nm using heat inactivated sample as blank (*ε* = 15 cm^2^ *μ*mol^−1^) [41]. One unit (U) of lipolytic activity was defined as the amount of enzyme that liberates 1 *μ*mol of 4-nitrophenol (pNP) per minute per mL. The specific lipolytic activity (U mg^−1^ protein) was calculated. Each sample was tested in triplicate.

### 2.6. Protein Determination

Protein content was determined according to the Lowry method [42] using BSA as standard.

### 2.7. Calculations

The following calculations were considered:

(1) foamate  rate  (mL min⁡−1)  =volume  (mL)  of  foamatetime  (min⁡)  to  fractionate,protein  recovery  rate  (mg min⁡−1)  =  protein  (mg)  in  foamatetime  (min⁡)  to  fractionate,lipolytic  activity  recovery  rate  (U min⁡−1)  =  activity  (U)  in  foamatetime  (min⁡)  to  fractionate,protein  recovery  (%)    =mass  (mg)of  protein  in  foamatemass  (mg)  of  protein  in  initial  solution×100,lipolytic  activity  recovery  (R)  (%)    =total  activity  (U)  in  foamatetotal  activity  (U)  in  initial  solution×100,protein  enrichment  ratio  =protein  (mg mL−1)in  foamateprotein  (mg mL−1)  in  initial  solution,lipolytic  activity  enrichment  ratio  (E)  =activity  (U mL−1)  in  foamateactivity (U mL−1)  in  initial  solution,purification  factor  (P) =specific  activity  (U mg−1  protein)  of  foamatespecific  activity  (U mg−1  protein)  of  initial  solution.

## 3. Results and Discussion

### 3.1. Influence of the Medium Composition, and pH and the Incubation Temperature and Time on the Extracellular Lipolytic Activity of *M. phaseolina*

**Figure 1:**
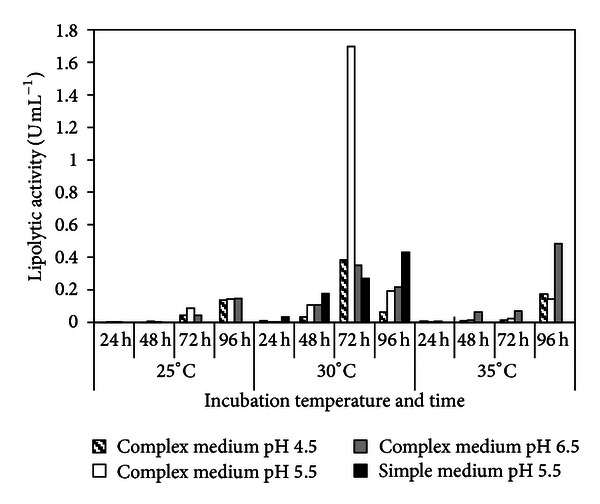
Extracellular lipolytic activity of *Macrophomina phaseolina* cultivated in complex medium (mineral salts, proteose peptone and olive oil) and simple medium (mineral salts and Tween 80), incubated at various pH and temperatures.

Extracellular lipolytic activity of *M. phaseolina* cultivated in complex and simple media is shown in Figure 1. 

In the complex medium at 25°C and 35°C, activity increased with time of incubation at the three levels of pH, reaching its maximum after 96 h of incubation. However, at 30°C, peak activities at the three pH levels were reached 24 h earlier, and the activity at pH 5.5, 1.7 U mL^−1^, was 400% higher than at the other two pH levels. When cultivated in mineral salts medium with Tween 80 at 30°C, pH 5.5, activity showed a steady increase during the whole cultivation period, reaching maximum (0.43 U mL^−1^) after 96 h of incubation. 

### 3.2. Foam Fractionation of Culture Supernatant from *M. phaseolina* Cultivated in the Complex Medium

Proteose peptone was among the components of the complex medium, and, due to the short period of cultivation (72 h), it possibly was not completely consumed by the fungus. Thus, it was probably present in a nonnegligible amount in the culture supernatant that was foam fractionated. Being amino acid polymers, proteoses and peptones are amphiphiles and present surface activity when in aqueous solution in contact with air. The basis for the foam fractionation of a mixture of amino acid polymers is the difference in their physicochemical behavior at the bubble surface [33], for example, differences in their surface activity. Solutes presenting higher activity are preferably adsorbed to those of lesser activity and, thus, become concentrated in the foamate. 

Bubbling air into the plain supernatant from* M. phaseolina* cultivated in the complex medium did not produce any ascending foam, probably due to the presence of lipid residues. Lipids can cause rupture of the bubble film through a Marangoni effect [43], and often present high surface activity and compete with proteins for the interfacial area even at relatively low concentrations, thus reducing the stability of protein-stabilized foams [44]. Triton X-100 (1.16 mM) was added to help solubilize lipid residue and stabilize foam. Foamate, obtained only at the maximum air flow allowed by the rotameter (20 L h^−1^), was collected for 2 min, after which the foam collapsed. Results for protein enrichment and lipolytic activity enrichment ratios and purification factor for this sample, as well as for the subsequent ones, are shown in Table 1. The foamate showed high protein enrichment ratio, but lipolytic activity enrichment ratio near 1, indicating that proteins other than the enzymes became concentrated in the foamate. Due to the high gas flow rate that created turbulence in the column, it is possible that the activity shown by this sample was due to the cosorption of the enzymes to the other protein species present in the supernatant and thus transferred to the foamate. No purification occurred.

To verify whether it was possible to obtain further fractionation after foam collapse, the air flow was reduced to 7.5 L h^−1^, the drainage bowl was removed, and the horseshoe bend was attached directly to the column. It allowed fractionating for further 27 min, and successive foamate samples were collected at no fixed time intervals. Compared to 20 L h^−1^, the first two foamate samples at 7.5 L h^−1^ (Table 1) showed decrease in protein concentration and no enrichment in lipolytic activity. Subsequent foamate at 7.5 L h^−1^ presented a decrease both in protein content and in activity. Fractionation preferably adsorbed protein species showing higher surface activity but no enzymatic activity. This is seen from the sharp decrease in the activity enrichment to a level well below 1, while the protein enrichment remained above 1. The removal of the drainage bowl and the relatively high air flow provided a reduced retention time in the column, not allowing for a good separation between contaminating proteins (proteoses and peptones) and the enzymes present in the supernatant. Thus, removal of the drainage bowl and reduction of the air flow to the minimum necessary to obtain foamate proved to be unsatisfactory for both protein enrichment and activity enrichment. Foam fractionation of the complex medium resulted in total recoveries of protein and lipolytic activity of 56% and 6%, respectively. Purification was negligible.

### 3.3. Foam Fractionation of Culture Supernatant from *M. phaseolina* Cultivated in the Simple Medium

To avoid the presence of proteins of exogenous origin as well as lipid residues in the supernatant, *M. phaseolina* was cultivated in a simple medium consisting of mineral salts. As carbon source and stimulant for the production of extracellular lipolytic enzymes, Tween 80 was added.

**Figure 2:**
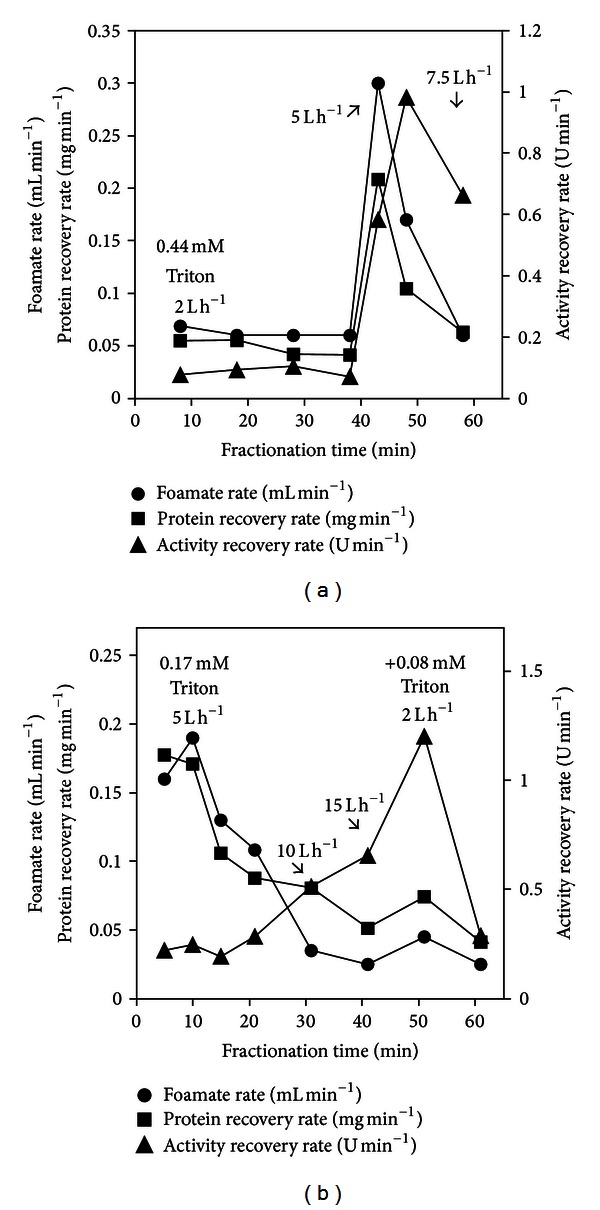
Foamate rate (circles), protein recovery rate (squares) and lipolytic activity recovery rate (triangles) during foam fractionation of culture supernatant from *Macrophomina phaseolina* cultivated in the simple medium. (a) = Triton 0.44 mM. (b) = Triton 0.17 mM + 0.08 mM after 50 min.

Similarly to the complex medium, no ascending foam was produced by the plain supernatant, even at the maximum air flow, 20 L h^−1^. Two experiments were then devised to determine the influence of different concentrations of Triton X-100 on the foam fractionation of this supernatant. In the first experiment, 0.44 mM was added, and three different air flow rates (2.0, 5.0, and 7.5 L h^−1^) were tested in sequence during 40, 10, and 10 min, respectively, on the same supernatant sample. Foamate samples were collected at fixed time intervals. Each change in air flow was preceded by foam collapse at the previous air flow rate.  Figure 2(a) shows the results of the kinetic parameters studied—foamate rate, protein recovery rate, and lipolytic activity recovery rate of the samples along the elapsed time during the fractionation process. 

At 2 L h^−1^, all three parameters were low, and the foamate rate was around 60 *μ*L per minute. After foam collapse at 2 L h^−1^, air flow rate was set to 5 L h^−1^ to obtain further foamate, and initially all three parameters increased, especially the activity recovery rate, which showed a 500% increase. However, the next sample collected at this air flow showed decrease in foamate and protein recovery rates. Surprisingly, however, the activity recovery rate almost doubled. The enhancement of this activity recovery at the expense of other protein molecules might result from the reduced concentration of inert proteins, which were foamed out of the system during the previous phase. After this second sample at 5 L h^−1^, foam collapsed again. At further increase in air flow, 7.5 L h^−1^, only one sample was obtained. It showed further decrease in the three parameters, foamate and protein recovery rates returning to levels similar to those at 2 L h^−1^, but activity recovery rate still at 0.66 U min^−1^. The experiment was terminated at this point.

The reasoning behind the second experiment was based on results obtained in the first one, namely, the effect of Triton X-100 on the selective fractionation of the inert proteins at the initial low air flow. As Triton itself is a contaminant, it is desirable to keep its concentration as low as possible. Thus, the effect of a reduced initial concentration in Triton, along with the effect of further addition of very small quantities of the surfactant, was investigated. The initial concentration was 0.17 mM, 60% less than in the previous experiment. 

Foaming processes are very sensitive to gas flow [33, 45]. Similar to the first experiment, in the second experiment, air flow rates of 5.0, 10.0, 15.0, and 2.0 L h^−1^ were tested in sequence during 20, 10, 10, and 20 min, respectively, and each change in air flow was preceded by a foam collapse. Before the last change in air flow, 0.08 mM Triton X-100 was added to the system. Due to the initial smaller quantity of Triton used in this second experiment, the lowest air flow rate that enabled foamate formation was 5 L h^−1^, 2.5 higher than the initial air flow in the first experiment.  Figure 2(b) shows the results of the kinetic parameters foamate rate, protein recovery rate, and lipolytic activity recovery rate of the samples along the elapsed time during the fractionation process. As expected, in this second experiment, the initial foamate rate was much higher than in the previous experiment (Figure 2(a)), as higher gas flow rates produce wetter foams due to reduced residence time in the column and less drainage. Foamate rate decreased over time, despite increase in the air flow. However, it did show a slight increase when additional Triton X-100 was introduced into the system, an effect that did not persist with further foaming. Protein recovery rate followed this same pattern. Lipolytic activity recovery rate was very low at the initial air flow, doubling when the gas flow was set at 10 L h^−1^ and increasing further at 15 L h^−1^. However, a sharp increase in activity recovery rate was noticed when the additional Triton X-100 was introduced, reaching 1.2 U min^−1^. The effect did not persist, and with further foaming, the activity recovery rate descended to the initial levels.

**Figure 3:**
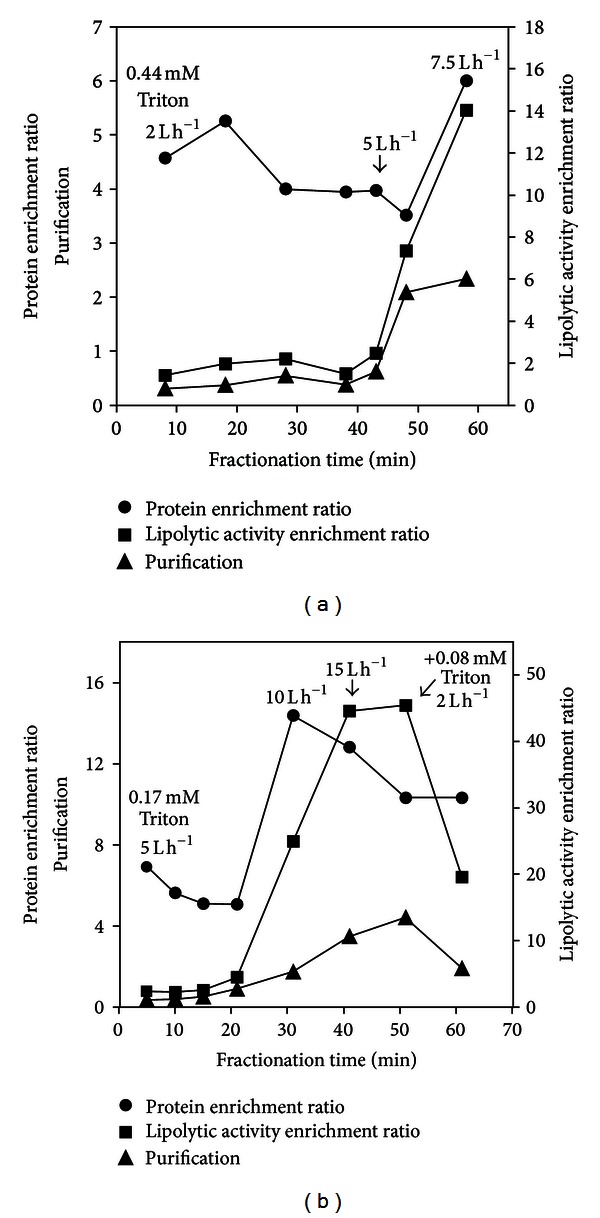
Protein enrichment ratio (circles), lipolytic activity enrichment ratio (squares) and purification factor (triangles) during foam fractionation of culture supernatant from *Macrophomina phaseolina* cultivated in the simple medium. (a) = Triton 0.44 mM. (b) = Triton 0.17 mM + 0.08 mM after 50 min.

Figure 3 shows the efficiency parameters—protein enrichment ratio, lipolytic activity enrichment ratio, and purification factor of the foamate samples along the time of the fractionation process for both experiments. In the first experiment, seen in Figure 3(a), at 2 L h^−1^ air flow, the protein enrichment ratio was high but lipolytic activity enrichment remained low (between 1.5 and 2), probably because the inert proteins were more surface active than the lipolytic enzymes, adsorbing faster and firmer to the bubble film. Differences in surface activity derive from differences in the molecule surface characteristics, like the tridimensional conformation, the electrostatic balance, and hydrophobic properties of the protein. In addition, it was shown that the composition of a mixed-protein film is kinetically driven and primarily controlled by the rate of arrival of each protein at the interface and the area available at the interface for molecular adsorption. A later-arriving protein, even if showing greater affinity for the interface than the first-arriver, could not displace it from the surface [46]. Lipases and esterases, extracellular lipolytic enzymes commonly produced by microorganisms, are macromolecules mainly of globular shape, demanding bigger areas at the interface to be able to adsorb. Besides, due to their size, their movement in the liquid might be hindered by the smaller-sized proteins and other macromolecules possibly present. These phenomena could have contributed to the selective removal of the inert proteins first at the initial low gas flow rate. As fractionation proceeded, Triton X-100 was steadily removed from the system with the successive foamate samples, and the bulk solution became increasingly depleted in it. The low molecular weight surfactant has 100% affinity to the interface, which causes its high rate of adsorption [47], and thus foams out first leaving the kinetically slower macromolecules behind. As consequence, the proteins still present in the bulk solution, among them the enzyme macromolecules, form an increasingly cohesive viscoelastic film at the air-water interface, increasing the film's viscosity, which demands a higher air flow to generate ascending foam. 

After collapse of the foam at 2 L h^−1^, the increase of air flow to 5 L h^−1^ caused additional decrease in the protein enrichment ratio. When the flow was altered to 7.5 L h^−1^ a sharp increase in the sample's protein content was noticed, reaching the highest level obtained in the experiment. The lipolytic activity enrichment ratio was negligible at 2 L h^−1^, showing an average of 1.8, increasing sharply at 5 L h^−1^ and even further at 7.5 L h^−1^, at the latter air flow reaching enrichment of 14. The high protein content and low activity of the foamate samples collected at 2 L h^−1^ resulted in no purification. However, at the 5 L h^−1^ air flow, the purification factor doubled, reaching 2.1, due to the threefold increase in the lipolytic activity without increase in protein content. The only sample obtained at 7.5 L h^−1^ air flow showed the highest purification factor, 2.3, and after foam collapsed, the experiment was terminated.

In the second experiment, the lowest air flow to produce foamate was 5 L h^−1^. Protein enrichment ratios (Figure 3(b)) of the samples obtained at this initial air flow rate were higher than those obtained with the initial air flow in the first experiment (Figure 3(a)). Both experiments showed a steady decrease along time in protein enrichment ratio at the initial air flow rate. The reason for this enhanced protein enrichment ratio at the initial gas flow in the second experiment is that higher flow rates produce more bubbles, which results in increased area available for protein adsorption. In addition, larger bubbles are obtained with frits of higher porosity, as in the present case, and as mentioned elsewhere [30, 31, 48], larger bubbles favor higher enrichments due to lesser liquid holdup and greater drainage. Doubling the gas flow to 10 L h^−1^ caused a sharp increase in protein enrichment. However, further increase in air flow or addition of Triton X-100 did not prevent the protein enrichment ratio from decreasing over fractionation time.

With an average of 2.9, the lipolytic activity enrichment ratio (Figure 3(b)) of the samples obtained at the initial air flow rate was double of that obtained at the initial flow rate in the first experiment (Figure 3(a)), also repeating the pattern of steady ratio during this phase of foaming. After the twofold and threefold increases in air flow rate in this second experiment, samples showed a very small volume, around 300 *μ*L collected over 10 min foaming, and appeared viscous. As Figure 3(b) shows, these two samples collected at 10 and 15 L h^−1^ enriched big amounts of protein, among them the lipolytic enzymes. Activity enrichment ratio obtained at 15 L h^−1^ was 44.6. As mentioned earlier, increased flow rates result in increased area available for adsorption, and more proteins, in the present case the lipolytic enzymes, are carried to the foamate.

After no further foamate was obtained at these air flow rates, a small amount of Triton X-100, 0.08 mM, was added to the bulk solution, gas flow was reduced to the minimum, 2 L h^−1^, and two additional foamate samples were obtained. The experiment was terminated at this point. 

Figure 2(b) shows that this increased the foamate rate, protein recovery rate, and activity recovery rate, probably due to the decrease in the foam's viscosity. However, the last sample, collected around 60 min, showed a big decrease in the activity recovery rate. Figure 3(b) shows that the foamate collected around 50 min showed an activity enrichment ratio only slightly superior to that of the previous sample despite its increase in activity recovery rate (Figure 2(b)). That is, more activity was recovered per time unit, however, with a concentration (U mL^−1^) very similar to the previous sample. The effect did not persist and the last sample showed a stable content in protein (Figure 3(b)) but a reduced content in enzyme. The increased recovery of the enzyme with the second addition of Triton can be explained by the fact that protein concentration in the bulk solution was decreasing, as seen from the steadily diminishing protein recovery rates, even at higher air flow rates. The initial foamate contained mostly inert proteins, and when it became necessary to increase air flow, the bulk solution was enriched in the lipolytic enzymes. Thus, proteins fractionated at these higher air flows were mainly lipolytic enzymes. However, protein content in the bulk solution after fractionation at 15 L h^−1^ was not enough to allow the formation of a stable foam and further foaming. When the small amount of additional Triton was introduced into the system, it enabled the creation of a flexible film due to cosorption of Triton and enzyme at the air-liquid interface [49]. The flexible film could now be foamed, enriched in lipolytic enzymes. Again, as foaming proceeded, Triton became depleted to the extent that activity recovery rate of the last sample fell dramatically (Figure 2(b)). 

As seen from Figure 3(b), the purification factor started to reach values above 1.5 only after the air flow was increased above 5 L h^−1^. The addition of Triton helped to increase purification by 26% over the previous sample, and it reached a factor of 4.4.

Table 2 shows the estimated protein recovery (%) and enrichment ratio, lipolytic activity recovery (*R*) (%) and enrichment ratio (*E*), and the purification factor (*P*), if all the samples collected had been pooled in one foamate sample. It also shows the same calculations for the set of samples in each experiment that showed the best results in *E* and *P*, that is, in the first experiment, only the samples collected at 5 L h^−1^ and 7.5 L h^−1^ air flow rates, and in the second experiment those collected at 10 L h^−1^, 15 L h^−1^ and 2 L h^−1^ flow rates.

From both experiments, it becomes evident that the overall estimated activity recovery and purification factor obtained with foam fractionation using air and a P 2 frit are not as high as those obtained with P 3 frits and nitrogen for lipase from *Pleurotus sapidus* (*R* = 95%, *P* = 11.6) [35] and *Fusarium* spec. (*R* = 93.6%, *P* = 5.9) [24] and esterases from *Pleurotus sapidus* (*R* = 89.3%, *P* = 16.0) [39]. However, foam fractionation of cutinase from *Coprinopsis cinerea* with a P 3 frit and air obtained *R* = 79%, *E* = 10.5, and *P* = 2.5 [33], results not too far from the present ones. 

Nevertheless, here is demonstrated the possibility to foam fractionate using the combined effect of air flow rate and use of surfactant as means to separate protein species. This is most evident in the second experiment. Different from other studies [33], in which activity recovery fell from 80% to 6% and purification factor from 4.3 to 1.1 when 0.2% Tween 80 was added to the solution in the column, it also shows that the addition of very small quantities of surfactant helps to increase recovery of the enzyme. By foaming out contaminating proteins first and then adjusting gas flow and stepwise addition of surfactant, it was possible to separately collect highly enriched foamate. Future work using a P 3 frit (porosity 16 to 40 *μ*m), a larger drainage bowl as suggested elsewhere [35], and the adjustment of air flow to the strategy here devised could improve the recovery of the lipolytic enzymes.

### 3.4. Lipolytic Activity of Foamate Samples

Samples collected at 50 and 60 min in the first experiment (Figure 3(a)) and at 40 and 50 min in the second (Figure 3(b)), which showed the highest purification factors, were tested on rhodamine B agar. According to the literature [50–52], lipases are carboxylesterases that hydrolyze triacylglycerols, for example, olive oil. Only the sample collected at 50 min of the second experiment (additional Triton used) showed a visible halo indicating a hydrolytic activity over normal chain triglycerides. It is possible that the other samples were not sufficiently concentrated to be able to show a positive result on rhodamine *B* test.

## 4. Conclusions

In summary, purification of lipolytic enzymes by foam fractionation was easier and more efficient in supernatant from Macrophomina phaseolina cultivated in a simple medium. Despite the long time taken in the foaming process with air, good recovery of lipolytic activity was attained and foamate contained lipases active on olive oil. By combining stepwise addition of surfactant and variable air flow rates, a new approach to foam fractionation could be devised. Advantages were the use of an easily available gas source and the reduced use of surfactant.

## Figures and Tables

**Table 1:** Protein enrichment ratio, lipolytic activity enrichment ratio, and purification factor of foamate during foam fractionation with 20 L h^−1^ and 7.5 L h^−1^ of culture supernatant from* Macrophomina phaseolina* cultivated in the complex medium.

Air flow	7.5 L h^−1^*							
	1**	2	3	4	5	6	7	8
Protein enrichment ratio	5.89	5.00	2.44	1.55	1.22	1.78	1.44	1.22
Lipolytic activity enrichment ratio	0.92	0.94	0.95	0.21	0.11	0.02	0.07	0.02
Purification factor	0.16	0.19	0.39	0.13	0.09	0.01	0.05	0.02

*Without drainage bowl.  **Sample collected at 20 L h^−1^with drainage bowl.

**Table 2:** Estimated protein recovery (%) and enrichment ratio, lipolytic activity recovery (%) and enrichment ratio, and purification factor of foam fractionation of the culture supernatant from *Macrophomina phaseolina* cultivated in a simple medium.

		1st experiment		2nd experiment	
		Pool^a^	Samples^b^	Pool^a^	Samples^c^
Protein recovery	%	24	13	33	15
Activity recovery	%	23	19	54	45
Protein enrichment ratio		4.3	4.2	7.6	11.9
Activity enrichment ratio		4.2	6.2	12.4	34.7
Purification factor		0.98	1.5	1.6	2.9

^a^Calculated for all samples taken together. ^b^Calculated for the samples collected at 5 L h^−1^ and 7.5 L h^−1^ air flow rates taken together. ^c^Calculated for the samples collected at 10 L h^−1^ 15 L h^−1^ and 2 L h^−1^ air flow rates taken together.
